# Changes in Abdominal Artery Diameter in Patients Treated for Acute Aortic Dissection

**DOI:** 10.3390/jcdd12040129

**Published:** 2025-04-02

**Authors:** Marian Burysz, Radosław Litwinowicz, Mariusz Kowalewski, Jerzy Walocha, Jakub Batko

**Affiliations:** 1 Department of Cardiac Surgery, Regional Specialist Hospital, 86-300 Grudziądz, Polandradek.litwinowicz@gmail.com (R.L.); 2 CAROL—Cardiothoracic Anatomy Research Operative Lab, Department of Cardiovascular Surgery and Transplantology, Institute of Cardiology, Jagiellonian University Medical College, 31-008 Kraków, Poland; 3 Thoracic Research Centre, Collegium Medicum, Nicolaus Copernicus University, Innovative Medical Forum, 85-067 Bydgoszcz, Poland; 4 Faculty of Medicine, Bydgoszcz University of Science and Technology, 85-796 Bydgoszcz, Poland; 5 Department of Cardiac Surgery, Central Clinical Hospital of the Ministry of Interior, Centre of Postgraduate Medical Education, 02-507 Warsaw, Poland; 6 Cardio-Thoracic Surgery Department, Heart and Vascular Centre, Maastricht University Medical Centre, 6229 HX Maastricht, The Netherlands; 7 Department of Anatomy, Jagiellonian University Medical College, 31-008 Kraków, Poland

**Keywords:** mesenteric ischemia, frozen elephant trunk, acute aortic dissection, anatomy

## Abstract

Background: Mesenteric ischemia significantly increases intraoperative mortality in patients with acute aortic dissection (AAD). The arterial diameter affects both blood flow and arterial resistance. There are no data in the literature on changes in arterial diameter in patients with AAD. It has already been demonstrated that changes in arterial diameter can be observed in patients with non-occlusive intestinal ischemia. The aim of this study was to compare the arterial branches of the abdominal aorta in patients with AAD preoperatively and postoperatively. Methods: Preoperative and postoperative contrast-enhanced computed tomography scans of 25 patients who had undergone the frozen elephant trunk procedure for the treatment of AAD were reconstructed and retrospectively analyzed with detailed medical data of the patients. Results: In patients without AAD at the level of the abdominal aorta, statistically significant differences were observed when comparing the diameter of the superior mesenteric artery (*p* < 0.001) and the renal arteries (*p* < 0.001) between preoperative and postoperative scans. Occlusion of the inferior mesenteric artery was more common in patients with AAD involving the abdominal aorta. Statistically significant differences in true and false lumen were observed at each level of the abdominal aorta after a successful frozen elephant trunk procedure. Conclusion: Significant changes in visceral artery diameter were observed at the abdominal aortic level in patients both with and without aortic dissection. Chronic or non-occlusive mesenteric ischemia may be associated with a lack of adjustment in arterial diameter. Patients with AAD of the abdominal aorta are more susceptible to occlusion of the inferior mesenteric artery.

## 1. Introduction

Acute aortic dissection is one of the most fatal diseases associated with the cardiovascular system. With an estimated prevalence of up to 6 cases per 100,000 people per year and a 48-hour mortality of up to 24% in treated patients, it remains one of the greatest challenges for cardiac surgery [1]. Despite the high early mortality, patients are prone to tangible complications, including mesenteric ischemia [2]. Its prevalence is not yet fully understood and varies between 3.7 and 15% in patients with type A acute aortic dissection [3,4]. It often coexists with spinal cord ischemia, acute renal failure, and limb ischemia. Mesenteric ischemia significantly increases intraoperative mortality in patients with acute aortic dissection (from 24% in the general population to 63.2% in patients with mesenteric ischemia), but surgical or hybrid management of acute aortic dissection significantly increases the chance of survival (odds ratio = 0.1) [3].

Despite the typically described types of causes of mesenteric ischemia, including the aortic type (caused by compression of the true lumen of the proximal aorta, reducing blood flow to the superior mesenteric artery, or its obstruction by an intimal flap) and branch type (caused by compression of the true lumen in the superior mesenteric artery by a false lumen), non-occlusive intestinal ischemia has been described in patients with acute aortic dissection, the occurrence of which is not related to the anatomy of the false lumen or the extent of the dissection process [5,6].

Contrast-enhanced computed tomography is a gold standard for the preoperative evaluation of patients with acute aortic dissection. It allows a precise assessment of the dissection localization and its impact on the aortic branches and is crucial for preoperative planning [7]. In addition, the diameter of the individual aortic branches can be assessed.

The arterial diameter affects both blood flow and arterial resistance. It is regulated by both the neuronal and chemical signals that lead to vasodilation or vasoconstriction. There are no data in the literature on changes in the arterial diameter of patients with acute aortic dissection, which plays a crucial role in preventing fatal complications or, if not properly regulated, may lead to their development. It has already been demonstrated that changes in arterial diameter can be observed in patients with non-occlusive intestinal ischemia [8]. Furthermore, in patients with continuous computed tomography scans, non-occlusive intestinal ischemia was shown to be associated with a reduced diameter of the superior mesenteric artery compared to measurements in previous imaging [9].

To date, there is no evidence of a relationship between changes in the arteries supplying the abdominal organs and the occurrence of mesenteric ischemia. We hypothesize that an assessment of the abdominal aortic branches pre- and postoperatively may play a role in the detection of patients with acute aortic dissection and mesenteric ischemia, particularly in patients who do not present with clinical signs of this condition on admission.

The aim of this study was to compare the arterial branches of the abdominal aorta in patients with acute aortic dissection pre- and postoperatively.

## 2. Materials and Methods

The authors of this study take full responsibility for the accuracy and integrity of the research and have taken the appropriate measures to ensure that any concerns about the work are addressed. This study was conducted in accordance with the ethical principles outlined in the Declaration of Helsinki (as revised in 2013). This study received approval from the Jagiellonian University Ethics Committee (NO.: 1072.6120.48.2022), and the need for individual informed consent was waived for this retrospective analysis.

In this retrospective study, we analyzed patients in whom the frozen elephant trunk procedure was used to treat acute aortic dissection. A description of the procedure can be found in our previous publication [10]. The inclusion criteria were as follows: no preoperative mesenteric ischemia or history of mesenteric ischemia, acute aortic dissection as an indication for the procedure, no previous aortic interventions, and contrast-enhanced computed tomography of the aorta being performed preoperatively and postoperatively.

### 2.1. Imaging Characteristics and Analysis

Contrast-enhanced computed tomography was performed with a 64-row dual-source scanner (Briliance 64, Philips, 595 Miner Rd., Cleveland OH, USA). Contrast enhancement was achieved in each patient with 1 mL of iodine contrast medium per kilogram. An image matrix of 512 × 512 pixels was used to reconstruct the images. The slice thickness and gradation were 1 mm and 0.5 mm, respectively. The computed tomography scans were analyzed on a dedicated workstation (Dell, Round Rock, TX, USA) using the advanced reconstruction and analysis software Mimics Innovation Suite 24.0 Software (Materialize, Plymouth, MI, USA). Measurements were performed by two separate researchers with at least 5 years of experience in analyzing contrast-enhanced computed tomography scans with virtual calipers. The mean of the measurements was reported. If the difference between the individual measurements was greater than 10%, the measurement was repeated by each researcher.

### 2.2. Visualizations and Measurements

**Figure 1:**
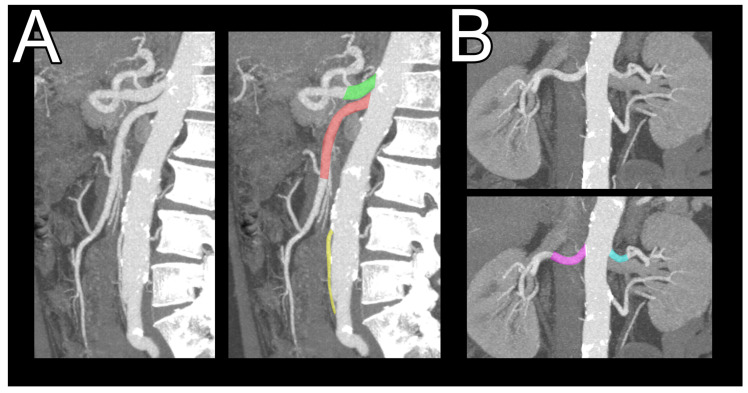
The examined arteries are shown on a multiplanar reconstruction with maximum intensity projection. (**A**) Celiac trunk—marked in green, superior mesenteric artery—marked in red, and inferior mesenteric artery—marked in yellow. (**B**) Left (marked in dark blue) and right (marked in pink) renal arteries. An additional (unmarked) renal artery can be seen. These accessory arteries were excluded from the analysis.

The entire aorta and its branches (including the left and right coronary artery, superior mesenteric artery, inferior mesenteric artery, renal arteries, and celiac trunk) were reconstructed in each study group using multiplanar reconstruction (Figure 1).

The diameter of each artery, 2 mm from the origin, was measured preoperatively and postoperatively in the acute aortic dissection group using computed tomography. If an additional renal artery was observed, only the main artery (defined as the one with the largest diameter) was measured. Aorta true lumen and false lumen were measured on the level of each artery origin. The artery origin from true or false lumen was evaluated.

Additionally, sub-analysis of patients with acute dissection located within the abdomen with a number of arteries originating from the false lumen, true and false lumen diameter changes, and a comparison of the abovementioned parameters of the population without abdominal dissection were performed.

### 2.3. Statistical Analyses

The collected data were further analyzed using IBM SPSS Statistics 29.0 software (Predictive Solutions, Pittsburgh, PA, USA). The distribution of the data was analyzed using the Shapiro–Wilk test. The direct comparison of continuous data between the study and control groups was performed using the U-Mann–Whitney test and the comparison of continuous data in the study group was performed using the Wilcoxon test. A comparison of categorical variables was performed with the chi-square test, or with the Fischer exact test if the population of the subgroup was smaller than 5. Data in this manuscript were expressed as a number with percentage and median with interquartile range, first, and third quartiles, respectively. A *p*-value of 0.05 was considered statistically significant.

## 3. Results

### 3.1. Characteristics of the Patients

The average age of the patients was 60 (53–67) years and 60% of the patients were male. Most patients were overweight, with a mean body mass index of 28.7 (24.1–30.8) kg/m^2^, hypertensive (*n* = 23, 92%), and smokers (*n* = 24, 96%), with a low rate of diabetes (*n* = 3, 12%). All patients enrolled in this study had atherosclerosis. Calcification of the aorta at any site was found in 60% of patients. Dyslipidemia was observed in 72% of patients. Hyperuricemia was observed in 16% of patients. Stanford type A dissection was seen in 20 (80) cases and Stanford type non-A non-B dissection was seen in 5 cases. Details on the affected aorta can be found in Table 1. The median operation time was as follows: 300 (270–465) minutes operation time, 180 (150–290) minutes cardiopulmonary bypass time, and distal hypothermic circulatory arrest of 32 (30–40) minutes. The prosthesis length was 150 mm in 13 patients and 100 mm in the other patients. Two patients died within 30 days of surgery. The median length of hospitalization was 20 (15–26) days. Mesenteric ischemia and cardiovascular dysfunction were observed in one patient.

### 3.2. Comparison of the Preoperative and Postoperative Measurements of the Analyzed Arterial Diameters in Patients with Acute Aortic Dissection

Detailed measurements for each artery can be found in Table 2.

Occlusion of the inferior mesenteric artery was observed in seven patients. There were no signs of thrombosis or atherosclerosis in the lumen of the artery in those patients. Additionally, this occlusion was not performed during the surgical procedure. In these patients, a statistically significant difference was found in the preoperative and postoperative diameter of the superior mesenteric artery (7.4 (6.6–8.1) mm vs. 8.7 (7.4–9.4) mm, *p* < 0.001) and the celiac trunk (7.9 (7.3–8.4) mm vs. 8.4 (7.9–9.1) mm, *p* < 0.001). No such change was observed in a single patient who developed mesenteric ischemia.

**Figure 2:**
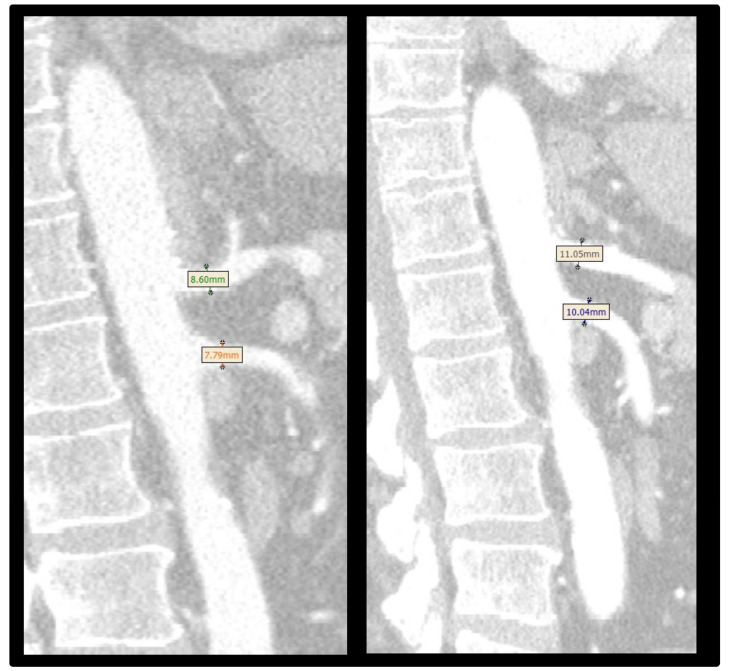
Changes in arterial diameter in patients with acute aortic dissection preoperatively (**left**) and postoperatively (**right**).

In patients without occlusion of the inferior mesenteric artery, there was a significant difference between the preoperative and postoperative diameter of the superior mesenteric artery (8.0 (7.2–8.2) mm vs. 8.6 (7.2–8.9) mm, *p* = 0.007), the diameter of the left renal artery (5.6 (5.3–6.0) mm vs. 6.1 (5.7–6.3) mm, *p* = 0.007), and that of the right renal artery (5.5 (5.2–5.9) mm vs. 6.3 (5.1–6.5) mm, *p* = 0.007) (Figure 2).

### 3.3. Comparison of Patients with Acute Aortic Dissection with and Without Involvement of the Abdominal Aorta

In eight patients (32.0%), the acute dissection involved the abdominal aorta. A detailed comparison between patients with and without abdominal aortic involvement in the acute dissection is shown in Table 3. Significant differences were observed between patients with acute aortic dissection at the level of the abdominal aorta pre- and postoperatively in the true aortic lumen dimeter at the level of the inferior mesenteric artery (*p* < 001). In patients without acute aortic dissection within the abdominal aorta, statistically significant differences were observed when comparing the diameter of the superior mesenteric artery pre- and postoperatively (*p* < 0.001) and the diameter of the renal arteries (left and right renal artery, *p* < 0.001).

In addition, the changes in the true and false lumen in patients with acute aortic dissection, including the abdominal aorta, were assessed at the level of the individual branches of the abdominal aorta (detailed measurements can be found in Table 4).

### 3.4. Sex Comparison of the Preoperative and Postoperative Measurements of the Analyzed Arterial Diameters in Patients with Acute Aortic Dissection

In the analyzed population, we observed larger preoperative diameters of each artery in the male population; however, this result was statistically significant only in the celiac trunk diameter (8.5 (7.8–9.1) mm vs. 7.4 (6.8–8.6) mm, *p* = 0.002) and the inferior mesenteric artery diameter (3.5 (3.0–3.7) mm vs. 4.1 (3.5–4.8) mm, *p* = 0.049). The occlusion of the inferior mesenteric artery was observed in four women and three men (40% female vs. 20% male, *p* = 0.28). Postoperative increase in the diameters in each analyzed artery was larger in males; however, this observation did not reach statistical significance for any of the analyzed arteries.

### 3.5. Abdominal Aortic Branch Adaptation in Patient with Mesenteric Ischemia

A 72-year-old female patient presented to our cardiac surgery department with a diagnosis of acute aortic dissection type A involving the aortic arch, which was confirmed by computed tomography. Prior to surgery, the patient reported severe chest pain. The patient’s BMI was 29.4 and Euroscore was 5.83. The patient was an active smoker, hypertensive, and had diabetes. The laboratory results showed decreased hemoglobin (11.5 g/dL), red blood cells (3.6 mln/uL), and hematocrit (34.1%), as well as elevated C-reactive protein (150 mg/L), cardiac troponin T (204 ng/mL), and N-terminal prohormone of brain natriuretic peptide (320 mU/L). A size-30/32/100 prosthesis was implanted. A CT scan was performed postoperatively, which confirmed the correct localization of the prosthesis. Postoperatively, 20 days after surgery, the patient developed chronic mesenteric ischemia. The patient underwent percutaneous revascularization of the inferior mesenteric artery and was in good condition during the one-year follow-up. At retrospective analysis, the lumen of the inferior mesenteric artery was occluded, and no changes in the diameter of the celiac trunk (7.1 mm vs. 6.9 mm) and superior mesenteric artery (6.8 mm vs. 6.9 mm) were noted pre- and postoperatively. The diameter of both renal arteries increased postoperatively (5.7 mm vs. 6.2 mm on the left side and 5.4 vs. 6.0 mm on the right side).

## 4. Discussion

### 4.1. Key Findings

Several findings should be emphasized when discussing the study results, as follows:

a. A total of 28% of patients had occlusion of the inferior mesenteric artery without evidence of thrombosis or atherosclerosis in the arterial lumen;

b. In a subgroup analysis, statistically significant differences were found between the preoperative and postoperative diameter of the celiac trunk and superior mesenteric artery in patients with inferior mesenteric artery occlusion;

c. A single case of mesenteric ischemia was observed in a patient with occlusion of the inferior mesenteric artery and there was no change in the diameter of the celiac trunk and superior mesenteric artery;

d. Occlusion of the inferior mesenteric artery was more common in patients with acute dissection of the abdominal aorta;

e. Statistically significant differences in the true and false lumen were observed at each level of the abdominal aorta after a successful frozen elephant trunk procedure;

f. The male population had a significantly larger preoperative diameter of the celiac trunk and inferior mesenteric artery.

### 4.2. Occlusion of the Inferior Mesenteric Artery in Acute Aortic Dissection—Major Clinical Impact or Insignificant Observation?

In our study, occlusion of the inferior mesenteric artery was a common finding that occurred more frequently in patients with abdominal aortic dissection. However, occlusion of the inferior mesenteric artery was observed in a single patient with mesenteric ischemia. To better understand the significance of this finding, the blood supply to the bowel should be discussed in detail.

The large and small bowel are supplied by three main arteries that arise from the abdominal aorta—the celiac trunk, the superior mesenteric artery, and the inferior mesenteric artery [11]. The aforementioned vessels are connected to each other by several anastomoses, including Moscovitz and Riolan arches, which form a bypass for blood supply in case of occlusion or reduced blood flow in one of the mesenteric arteries [11,12]. In addition, the marginal artery of Drummond directly connects the superior and inferior mesenteric arteries and provides a continuous blood supply to the colon [13]. In some cases, connections to other arteries in the abdomen are also observed [12,13]. The inferior mesenteric artery typically supplies the distal part of the transverse colon and the descending colon and rectum.

In daily clinical practice, however, various causes of occlusion of the inferior mesenteric artery can be observed. During the treatment of type 2 endoleaks originating from the inferior mesenteric artery or occurring during resection of a rectal carcinoma, the inferior mesenteric artery is sacrificed [14,15]. In these cases, however, we do not observe a dramatic increase in the prevalence of mesenteric ischemia [14,15]. A randomized study has shown that there is no difference in complications between high and low ligation of the inferior mesenteric artery in patients undergoing rectal cancer surgery. However, high ligation is more frequently associated with leakage through anastomoses, which provides important information about the blood supply to this region despite the occlusion of the inferior mesenteric artery [15]. In most cases, the additional blood supply from the superior mesenteric artery and celiac trunk is sufficient to compensate for the loss of blood supply caused by occlusion of the inferior mesenteric artery. It is important to understand that, despite an efficient collateral supply, mesenteric ischemia is more likely to occur if an additional factor occurs that restricts the blood supply from the anastomoses. This conclusion is supported by several studies in which patient age and a greater number of occluded arteries have been shown to be independent risk factors for mesenteric ischemia [16,17,18]. It should be noted that non-occlusive mesenteric ischemia typically occurs in patients with severe comorbidities or heart failure, which is consistent with the profile of patients with acute aortic dissection [5,9,16]. In addition, one patient in our study did not have a typical postoperative change in the diameter of the superior mesenteric artery and celiac trunk, which could lead to the restriction of blood flow to the bowel, due to the increased resistance and decreased blood flow volume directly related to this parameter. Other causes, such as preoperative constriction of blood flow, inadequate collateral circulation, or reperfusion syndrome, may have played a role in the development of chronic mesenteric ischemia in this patient. In patients with inferior mesenteric artery occlusion and acute aortic dissection, a significant increase in the diameter of the celiac trunk and superior mesenteric artery was observed, which is associated with increased blood flow through these vessels. This change could play an important protective role in the prevention of mesenteric ischemia.

In summary, patients with occlusion of the inferior mesenteric artery are at increased risk of potential mesenteric ischemia and should be evaluated for suspected mesenteric ischemia in the presence of uncharacteristic abdominal-related symptoms to protect them from potentially fatal complications, especially in patients with abdominal aortic involvement, in whom suspicion of further aortic dissection may distract from a correct diagnosis. In addition, patients without postoperative changes in the diameter of the celiac trunk or superior mesenteric artery should be followed up more frequently to detect possible mesenteric ischemia early.

### 4.3. Are Ischemic Complications Sex Specific?

According to our observations, the men had larger diameters of the individual artery preoperatively, with statistical significance for the celiac trunk and the inferior mesenteric artery. In addition, occlusion of the inferior mesenteric artery was observed more frequently in women, although this observation did not reach statistical significance. The postoperative increase in diameter in each artery studied was greater in men, but this observation did not reach statistical significance for any of the arteries studied. These results should be interpreted with caution, and a broader context of gender predisposition to the occurrence of mesenteric ischemia should be mentioned. Acute aortic dissection is more frequently diagnosed in men [19]. In addition, women with acute aortic dissection are typically older and often present later than men, both factors that increase the risk of mesenteric ischemia [19]. In Kase’s study, mesenteric ischemia was found to be more common in women (57% versus 43%) [20]. It should be mentioned that mesenteric ischemia can occur as a result of superior mesenteric artery thromboembolism, which is more common in women [21,22]. It is associated with several factors, including hormonal prothrombotic effects, pregnancy and oral contraception, and the anatomy of the superior mesenteric artery, which is more prone to mechanical occlusion due to the narrower angle between the aorta and its orifice in women [23]. In addition, it has been widely demonstrated that women are more often underdiagnosed, which invariably leads to fatal complications in the case of mesenteric ischemia [24]. Based on all of these arguments, it should be clear that ischemic complications are not gender-specific, but great caution should be exercised in female patients with risk factors for mesenteric ischemia.

### 4.4. Frozen Elephant Trunk—Is It Just the Treatment of the Aortic Arch?

Interestingly, the frozen elephant trunk procedure in patients with abdominal aortic dissection resulted in a decrease in the false lumen with a simultaneous increase in the true lumen after surgery. Our results are consistent with previously published studies [25,26]. This proves that hybrid treatment of aortic arch pathology has beneficial effects on the entire aorta. It should be noted that invasive treatment of acute mesenteric ischemia prior to the correction of aortic dissection in hemodynamically stable patients has no negative impact on the ability to perform the frozen elephant trunk procedure [27].

### 4.5. Exploring Potential Causes of Inferior Mesenteric Artery Occlusion

We hypothesize that the observed occlusion may be related to endothelial dysfunction, mesenteric steal syndrome, hypovolemia, or other mechanisms associated with acute aortic dissection [28,29]. The endothelial mechanism of arterial occlusion has been described in detail in the analysis of the causes of central retinal artery occlusion [30]. In addition, occlusion of the inferior mesenteric artery may be secondary to the dysfunction of other visceral arteries. In some cases, it is possible that the inferior mesenteric artery was already occluded before the acute aortic dissection occurred [14,15].

### 4.6. Changes in Abdominal Arteries Sheds New Light on Mesenteric Ischemia?

To understand the impact of changes in arterial diameter on mesenteric ischemia, a deeper explanation of this complication is needed. Mesenteric ischemia, both acute and chronic, is underdiagnosed and is associated with a high mortality rate [31,32,33].

Acute mesenteric ischemia is, in most cases, associated with occlusion or stenosis (in most cases, with a blood clot) of the superior mesenteric artery [16,20]. The prognosis depends on how much time elapses between the onset of ischemia, the intervention, and how early this condition is recognized [33]. In most cases, acute mesenteric ischemia is associated with severe abdominal pain, diarrhea or vomiting, and comorbidities, which increases the risk of thromboembolism [6,16,18]. Occlusion of the artery is associated with heart failure, dehydration, or hypercoagulation [6,16]. In most cases, patients with acute mesenteric ischemia require immediate intervention to survive [16,24]. The first intervention should be revascularization of the artery, followed by an assessment of the bowel, and possible resection. Both endovascular and open procedures should be considered, with the endovascular approach being associated with a lower number of complications and a lower mortality rate [34,35,36].

Chronic mesenteric ischemia is associated with occlusion of any mesenteric artery and the celiac trunk. Its incidence increases with age [37]. In addition, it should be noted that asymptomatic occlusion of one or more mesenteric arteries is present in almost 40% of patients with an abdominal aortic aneurysm [37]. Typical symptoms of chronic mesenteric ischemia are abdominal pain after eating, weight loss without loss of appetite, and defecation disorders [6,16]. There is no indication of a need for surgery in asymptomatic patients. In symptomatic cases, however, surgery should not be avoided, as this is associated with a worsening of the clinical condition and intestinal necrosis [38,39]. In most cases, endovascular stenting and angioplasty are the treatments of first choice. If treatment is unsuccessful, surgery can be performed [39].

Contrast-enhanced, high-resolution computed tomography angiography is required to confirm the diagnosis of mesenteric ischemia [40]. In patients with suspected mesenteric ischemia, computed tomography angiography plays a crucial role in the diagnosis, with a sensitivity of 71 to 96% and a specificity of over 90% [41,42]. Computed tomography has replaced angiography in these cases, as it is non-invasive. However, invasive methods are still used in patients in whom computed tomography does not provide clear results [43]. Computed tomography allows not only the assessment of the mesenteric arteries, but also the evaluation of the bowel wall, the presence of fluid in the peritoneum, and other pathologies associated with necrosis of the bowel.

In addition to the two types of mesenteric ischemia mentioned above, non-occlusive intestinal ischemia should also be mentioned [5]. This is a rare form of mesenteric ischemia that is not associated with either false luminal anatomy or extension of the dissection [5,9]. It is caused by primary mesenteric arterial vasoconstriction, which, in most cases, is caused by a spasm of the branches of the superior mesenteric artery [8,9]. It should be noted that arterial occlusion is not observed in this type of mesenteric ischemia. Correct identification of non-occlusive mesenteric ischemia is challenging because there are only non-specific changes in the arterial vessels of the bowel [5,8,9]. Furthermore, in patients with continuous computed tomography scans, non-occlusive bowel ischemia was shown to be associated with decreased superior mesenteric artery diameter compared to measurements at prior imaging [9].

According to our observations, the preoperative diameter of each of the abdominal arteries studied was smaller than in populations described in the literature [44,45,46,47,48]. Such persistent changes may lead to the development of fatal complications due to organ dysfunction. The treatment of acute aortic dissection in our study resulted in a significant increase in the diameter of the superior mesenteric artery, which may lead to recurrence of malperfusion.

It should be noted that such an adaptation does not play a decisive role in the mesenteric insufficiency caused by mechanical occlusion of the superior mesenteric artery or the celiac trunk. The inferior mesenteric artery is much smaller than the superior mesenteric artery, and it is very unlikely that it is able to supply the intestines, which are normally supplied by the superior mesenteric artery.

The changes in arterial diameters described in this study are particularly important for the early detection of patients at risk of late-onset mesenteric ischemia. Further evaluation of this phenomenon and its confirmation in a larger cohort of patients could lead to the use of treatment that supports adequate blood flow to the abdominal organs for prevention in patients without abdominal artery diameter changes.

### 4.7. Renal Artery Changes and Acute Aortic Dissection

Adaptation of the human body with the aim of maintaining homeostasis is one of the most important traits crucial for survival under unfavorable conditions. Our study sheds light on the arterial adaptation of the branches of the abdominal aorta, which is crucial for several reasons. Patients with acute aortic dissection are under great biological stress, which requires various systemic changes, including changes in the cardiovascular system. In the initial stage of dissection, increased arterial pressure and tachycardia are observed, the main cause of which is the maintenance of blood flow to the vital organs [49]. However, hypotension can be observed after decompensation [49]. This pathophysiological fact is represented in our study by the reduced diameter of the renal arteries. The reduction in diameter leading to temporary renal ischemia triggers the activation of the renin–angiotensin–aldosterone system, which rapidly increases systemic blood pressure [50]. After aortic repair, the renal arteries restore their standard diameter, because the risk factor that caused the increase in arterial blood pressure has been eliminated. Such adaptation, as observed in our study, can be controlled by both nervous and hormonal regulation and is crucial for patient survival [50]. However, it should be noted that hypertension can preexist and be the cause of aortic dissection, with great contribution to its progression. Additional factors, including systemic sympathetic activation, pain, hemodynamic changes, and complex neurohormonal regulation impact arterial blood pressure in patients with acute aortic dissection [49]. The described mechanisms, associated with renal ischemia, play additional roles in this process.

### 4.8. Limitations and Future Studies

Future studies should investigate whether the arterial adaptation described in this study can also be observed in patients with chronic aortic dissection and what impact this has on patient survival and complication rates. In addition, a larger cohort of patients should be studied, especially patients with chronic and non-occlusive mesenteric ischemia. In our population, most patients were hypertensive and had dyslipidemia. However, hyperuricemia was less frequently observed, although it is associated with the occurrence of acute aortic dissection [51,52]. It is known that the aforementioned comorbidities are associated with an increased risk of acute aortic dissection [53]. In our analysis, one patient with chronic mesenteric ischemia suffered from both dyslipidemia and hypertension. Future studies should investigate in detail the influence of these comorbidities on the occurrence of arterial adaptation and mesenteric ischemia. We omitted such an analysis in our study due to the limited sample size.

Some limitations should be noted. This is a retrospective analysis of a single center. Only 25 patients were included in this study. Only one patient with mesenteric ischemia was operated on in our center, so a direct comparison with this subgroup was not possible [54,55]. It was not possible to examine the vessels of each patient before the onset of acute aortic dissection. We did not analyze the impact of arterial changes on long-term complications or mortality.

## 5. Conclusions

Statistically significant changes in visceral artery diameter were observed at the level of the abdominal aorta in patients both with and without aortic dissection. A single case of mesenteric ischemia was observed in a patient with occlusion of the inferior mesenteric artery, and no change in the diameter of the celiac trunk and superior mesenteric artery was observed. Occlusion of the inferior mesenteric artery was more common in patients with acute dissection involving the abdominal aorta. Statistically significant differences in true and false lumens were observed at each level of the abdominal aorta after a successful frozen elephant trunk procedure.

## Figures and Tables

**Table 1:** Details of aorta involved in acute aortic dissection.

Parts of Aorta Involved	N (%)
Ascending + arch	17 (68.0%)
Ascending + arch + thoracic + abdominal	3 (12.0%)
Arch + thoracic + abdominal	5 (20.0%)

**Table 2:** Comparison of the analyzed arteries preoperatively and postoperatively.

	Preoperative AAD (n = 25)	Postoperative AAD (n = 25)	*p*
Left coronary artery diameter	4.5 (3.8–7.0)	4.6 (4.2–5.2)	0.61
Right coronary artery diameter	3.7 (3.6–4.1)	3.8 (3.5–4.2)	0.86
Celiac trunk diameter	8.3 (7.1–8.9)	8.0 (7.2–9.0)	0.55
Superior mesenteric artery diameter	7.9 (6.9–8.2)	8.5 (7.0–9.0)	0.50
Inferior mesenteric artery diameter	3.9 (3.4–4.6)	3.7 (3.5–4.6)	1.000
Left renal artery diameter	5.6 (4.9–6.1)	6.1 (5.7–6.6)	0.80
Right renal artery diameter	5.5 (5.2–6.0)	6.2 (5.1–6.5)	0.73

AAD—acute aortic dissection.

**Table 3:** Detailed comparison of patients with acute aortic dissection with and without involvement of the abdominal aorta.

	AAD in AA (n = 8)		AAD Apart from AA (n = 17)			
	Preoperative	Postoperative	Preoperative	Postoperative	P1	P2
Left coronary artery diameter	4.2 (3.9–7.1)	4.4 (4.1–6.6)	4.8 (3.9–6.4)	4.8 (4.3–5.1)	1.000	1.000
Right coronary artery diameter	3.7 (3.6–4.4)	3.7 (3.6–4.4)	3.9 (3.6–4.1)	3.8 (3.6–4.2)	0.932	0.887
Celiac trunk diameter	7.8 (7.5–9.2)	8.2 (7.4–10.6)	8.3 (7.8–8.7)	8 (7.3–8.8)	0.754	0.628
Superior mesenteric artery diameter	7.9 (7.1–8.3)	7.9 (7.3–9.3)	8 (7.2–8.2)	8.7 (7.2–8.9)	0.887	1.000
Inferior mesenteric artery diameter	3.7 (3.4–4.7)	3.2 (2.8–3.7)	4 (3.5–4.4)	3.7 (3.5–4.3)	0.932	0.871
Left renal artery diameter	5.4 (4.8–6.3)	5.9 (5.6–6.8)	5.7 (5.5–6)	6.1 (6–6.3)	0.549	0.754
Right renal artery diameter	5.4 (5.2–6.2)	5.4 (5–6.4)	5.5 (5.3–5.9)	6.3 (5.5–6.4)	0.669	0.262
Aortic arch diameter	41.1 (37.2–42.5)	30 (28–32)	40.5 (36.0–41.3)	30 (28–32)	0.872	1.000
Inferior mesenteric artery occlusion	4 (50%)	4 (50%)	1 (5.9%)	3 (17.6%)	0.010	0.092
Aortic true lumen on the level of inferior mesenteric artery	8.8 (2.2–14.6)	14.6 (8.0–16.7)	18.2 (16.5–21.3)	21.1 (20.8–23.2)	<0.001	<0.001

AA—abdominal aorta; AAD—acute aortic dissection; P1—*p*-value for comparison between preoperative characteristics; P2—*p*-value for comparison between postoperative characteristics.

**Table 4:** True and false lumen of the abdominal aorta at the level of each abdominal aortic branch in patients with acute aortic dissection involving the abdominal aorta.

	Preoperative AAD (n = 8)	Postoperative AAD (n = 8)	*p*
True lumen on the level of celiac trunk	6.7 (6.1–11.6)	12.8 (10.5–22.1)	<0.001
False lumen on the level of celiac trunk	20.0 (14.6–21.4)	16.9 (13.3–19.8)	<0.001
Celiac trunk origin in false lumen	1 (12.5%)	2 (25.0%)	0.465
Dissection flap entering celiac trunk lumen	4 (50.0%)	4 (50.0%)	1.000
True lumen on the level of superior mesenteric artery	6.4 (4.7; 20.3)	11.4 (6.6–19.7)	0.032
False lumen on the level of superior mesenteric artery	18.0 (16.3–20.6)	16.2 (8.0–19.8)	0.041
Superior mesenteric artery origin in false lumen	1 (12.5%)	1 (12.5%)	1.000
Dissection flap entering superior mesenteric artery lumen	2 (25.0%)	2 (25.0%)	1.000
True lumen on the level of inferior mesenteric artery	8.2 (2.6–20.8)	14.7 (8.8–20.8)	<0.001
False lumen on the level of inferior mesenteric artery	13.2 (7.2–22.4)	7.2 (6.4–20.1)	<0.001
Inferior mesenteric artery origin in false lumen	1 (12.5%)	1 (12.5%)	1.000
Dissection flap entering inferior mesenteric artery lumen	0 (0.0%)	0 (0.0%)	1.000
True lumen on the level of renal arteries	6.1 (3.5–18.3)	11.2 (9.0–22.2)	<0.001
False lumen on the level of renal arteries	16.2 (10.4–20.6)	14.1 (6.7–18.9)	<0.001
Left renal artery origin in false lumen	4 (50.0%)	4 (50.0%)	1.000
Dissection flap entering left renal artery lumen	2 (25.0%)	2 (25.0%)	1.000
Right renal artery origin in false lumen	4 (50.0%)	4 (50.0%)	1.000
Dissection flap entering left renal artery lumen	0 (0.0%)	0 (0.0%)	1.000

AAD—acute aortic dissection.

## Data Availability

Data are available from the corresponding author upon reasonable request.

## Abbreviations

The following abbreviations are used in this manuscript:

AAD	Acute aortic dissection
